# Control of Cutaneous Leishmaniasis Using Deltamethrin Treated Nets in Comparison to Indoors Residual Spraying in a Rural Area of Iran

**Published:** 2017-06

**Authors:** Mohammad Hassan KAYEDI, Yavar RASSI, Ali CHEGENI-SHARAFI, Reza ROSTAMI, Sayena RAFIZADEH, Nargess ABDALI

**Affiliations:** 1. Razi Herbal Medicines Research Center and Department of Parasitology, School of Medicine, Lorestan University of Medical Sciences, Khorramabad, Iran; 2. Dept. of Medical Entomology and Vector Control, School of Public Health, Tehran University of Medical Sciences, Tehran, Iran; 3. Disease Control Unit, Provincial Health Center, Lorestan University of Medical Sciences, Khorramabad, Iran; 4. Razi Herbal Medicines Research Center, Lorestan University of Medical Sciences, Khorramabad, Iran; 5. Ministry of Health & Medical Education, Tehran, Iran; 6. Student Research Committee, Lorestan University of Medical Sciences, Khorramabad, Iran; 7. North Research Center, Pasteur Institute of Iran, Amol, Iran

**Keywords:** Cutaneous leishmaniasis, Deltamethrin, Insecticide treated nets, Indoors residual spraying, Iran

## Abstract

**Background:**

Cutaneous leishmaniasis (CL) is an important vector-borne disease in Iran. The present study aimed to evaluate the efficacy of Deltamethrin treated nets in comparison to Indoors Residual Spraying (IRS) for the control of leishmaniasis in a new focal area of Iran.

**Methods:**

The study was carried out in 5 villages in the west of Iran during 2012–2013. One village was selected to the application of Insecticide Treated Nets (ITNs, 25 mg/m^2^ deltamethrin) among residents. Four other villages were considered for IRS using deltamethrin emulsion, 2.5% EG and Aicon 10WP. Spraying was repeated after 3 month. Other control methods e.g. environmental managements, and treatment of positive cases were carried out in all villages.

**Results:**

After 1 yr of trial, the cases of disease reduced from 50 to 2 and 128 to 5 for ITNs distributed nets and use of IRS in the villages, respectively.

**Conclusion:**

Reduction of CL cases in both methods were significant (*P*<0.001).

## Introduction

Cutaneous leishmaniasis (CL) is a zoonotic disease caused by *Leishmania* spp and vectors are sand flies. Transmission of this disease mainly occurs in the South America, North of Africa, Middle East, and central Asia. More than 90% of annual reports of this disease are reported from Brazil, Peru, Syria, Afghanistan, Saudi Arabia and Iran (1). This disease is one of the most important vector-borne diseases in some rural and urban areas in Iran. Annual cases of the disease have been reported in 17 of the 31 provinces (2, 3). Regarding the increase in population, new foci of the disease have emerged in some provinces across the southern parts of the Zagros Mountains and in central areas of the country (2, 4). One of these new focus areas on the southern slopes of the Zagros Mountain is Pole-Dokhtar, which is a county of Lorestan Province (5, 6). Table 1 shows positive cases and incidence of disease from 2004 to the end of 2015. There is no prophylaxis for this disease. Vaccines and different therapeutic methods are studied (7) and one of the disease control methods is using nets and avoiding biting.

**Table 1:** Positive cases and incidence (per 100000 population) of Cutaneous Leishmaniasis

**Year**	**Sarab-Hamam villages**		**Pole-Dokhtar county**		**Lorestan Province**	
	**Positive cases**	**Incidence of disease**	**Positive cases**	**Incidence of disease**	**Positive cases**	**Incidence of disease**
2004	0	0	3	3.83	32	1.88
2005	2	58.09	12	15.31	54	3.11
2006	166	4337.60	178	228.88	300	17.37
2007	8	198.61	23	29.17	137	7.71
2008	0	0	17	21.88	57	3.23
2009	0	0	31	39.01	82	4.64
2010	0	0	19	23.67	85	4.76
2011	0	0	11	13.70	71	3.91
2012	0	0	14	17.26	91	4.94
2013	0	0	14	17.30	106	5.76
2014	0	0	20	24.82	239	12.7
2015	21	478.03	117	145.20	324	17.23

Indoor Residual Spraying (IRS) is an important method for control of malaria and *Leishmania* vectors used in many countries (8, 9). Besides, insecticide-treated nets (ITNs) and wash resistant versions of them named long lasting insecticide-treated nets (LLITNs) are very effective tools against *Anopheles* spp and have been recommended by WHO (10–12). In recent years, some studies have shown the efficacy of ITNs and LLITNs in control of CL (2, 13–17). ITNs have reduced the incidence of disease (18–23).

The aim of the present study was to evaluate the efficacy of deltamethrin-treated nets in comparison to IRS to control transmission of CL in a new focal area of Iran.

## Materials and Methods

The present study was carried out in 5 villages of Sarab Hamam area in Pole-Dokhtar county, Lorestan Province in Western Iran during 2012–2013. The study population was 3827 people, including 816 families and 575 households. Regards to limited number of nets, one of the villages with 1420 inhabitants and 300 families were randomly considered to distribute ITNs between residents. The brand of ITNs was “attack” with dimensions of (190×180×160 cm), 156 Mesh and 100 Denier made of 100% polyester fibers. The “attack” nets were treated in factory with 25 mg/m^2^ deltamethrin using K-O thrin bayed from Bayer Company Germany. Two adults or three children can sleep under a net. The Company claimed that the efficacy of nets will keep at least 12 months after using or up to 3 washes with detergents.

Ethical approval was received from the Ethical Committee of the Lorestan University of Medical Sciences (No: P/15/2339; 27, June 2012).

In the beginning of transmission season of disease 620 “attack” nets were distributed among householders. Each family received two nets. In some cases, that number of inhabitants was more than five, three nets were given to each family. At the same time, 2 stick insect repellents (diethyl toluamide 33%) were given to each family (22). Investigators trained people that how and when they can use nets and repellents.

Four other villages with population of 2407 and 516 families were considered for IRS during the transmission season using deltamethrin Emulsion, 2.5% EG (20–25 cc in 20 L water) or Aicon 10WP, 2 sachet (62.5 gr) in 18–20 L water (active ingredient 25–30 mg/m^2^ of sprayed surface). All indoor and outdoor surfaces of animal and human dwellings in the villages were sprayed in the beginning of transmission season. Spraying was repeated after 3 months. Investigators emphasized that workers spray basement and holes in outdoor walls.

In all villages of the area, including ITNs distributed village, other control methods, such as transfer of rubbish and animal feces to the outside of the villages, burning of rubbish, active and passive finding of cases using laboratory diagnosis and treatment of positive cases were carried out.

Community-based programs were carried out in large scales for all people in the villages. Training programs were carried out in schools, mosques and Health center of the villages. Habitats, biology, and ecology of vector, transmission, diagnosis and prevention of disease were trained to the people by investigators. People have trained that use sticks insect repellents on naked parts of their bodies from evening.

To identify species of *Phlebotomus* sand flies and their seasonal and monthly activity in the area, 120 sticky traps were used to collect them from human and animal dwellings, outdoor shelters (mountain), outdoor walls and warehouses in the villages from May to Nov every month. The research team coated 120 A4 paper with castor oil and then transferred them to villages. Sticky traps were put in different places (24 for each place) at sunset and were collected the next day, before sunrise.

Sticky traps were sent to laboratory and sand flies were removed from oily papers to a Petri dish then Acetone solution was used to remove oil from body of sand flies and then were put in 70% ethanol, labeled and sent them for school of Public Health, Tehran University of Medical Sciences to identify species.

## Results

### Ecology and monthly activity of sand flies

Nine hundred and twelve *Phlebotomus*, 636 (69.74%) male and 276 (30.26%) female were collected. Nine species of *Phlebotomus* Genus were identified. The dominant species was *P. papatasi* (81.80%). *P. alexandri* (8.66%) and *P. sergenti* (3.84%) had highest population of *Phlebotomus* in the area respectively after *P. papat*asi (Table 2). One hundred and fifty-two *Sergentomyia*, 66 (43.42%) male and 86 (56.58%) female were collected. Six species of *Sergentomyia* genus were captured from the area. *S. dentata* (48.03%) and *S. clydei* (38.82%) were dominant species (Table 2).

**Table 2:** Genus and species of sand flies caught from villages

***Sergentomyia* Species**	**Male (%)**	**Female (%)**	**Total (%)**	***Phlebotomus* species**	**Male (%)**	**Female (%)**	**Total (%)**
*S.dentata*	32 (43.84)	41 (56.16)	73 (48.03)	*P.papatasi*	525 (70.38)	221 (29.62)	746 (81.80)
*S.clydei*	27 (45.76)	32 (54.24)	59 (38.82)	*P.sergenti*	29 (82.86)	6 (17.14)	35 (3.84)
*S.tiberiadis*	4 (36.36)	7 (63.64)	11 (7.24)	*P.alexandri*	59 (74.68)	20 (25.32)	79 (8.66)
*S.sintoni*	3 (50.00)	3 (50.00)	6 (3.95)	*P.elenorae*	0 (0.00)	6 (100.00)	6 (0.66)
*S.theodori*	0 (0.00)	2 (100.00)	2 (1.32)	*P.jacusieli*	0 (0.00)	3 (100.00)	3 (0.33)
*S.squamipleuris*	0 (0.00)	1 (100.00)	1 (0.66)	*P.major*	1 (100.00)	0 (0.00)	1 (0.11)
				*P.kazeruni*	3 (13.04)	20 (86.96)	23 (2.52)
				*P.halepensis*	2 (100.00)	0 (0.00)	2 (0.22)
				*P.mongolensis*	17 (100.00)	0 (0.00)	17 (1.86)
Total	66	86	152	Total	636	276	912

**Fig. 1:**
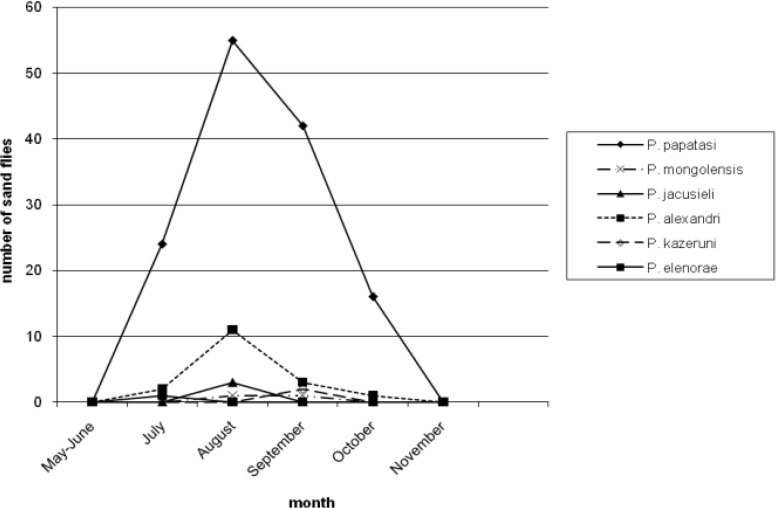
Monthly activity of *Phlebotomus* species from May to November caught from animal dwellings

**Fig. 2:**
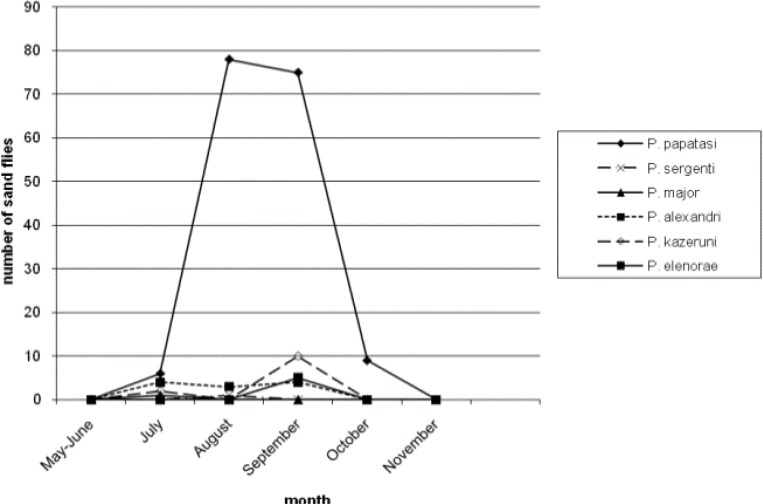
Monthly activity of *Phlebotomus* species from May to November caught from human dwellings

**Fig. 3:**
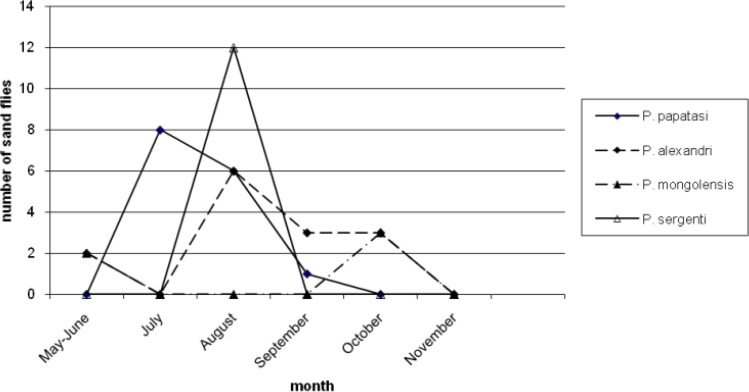
Monthly activity of *Phlebotomus* species from May to November caught from outdoor shelters (mountain)

**Fig. 4:**
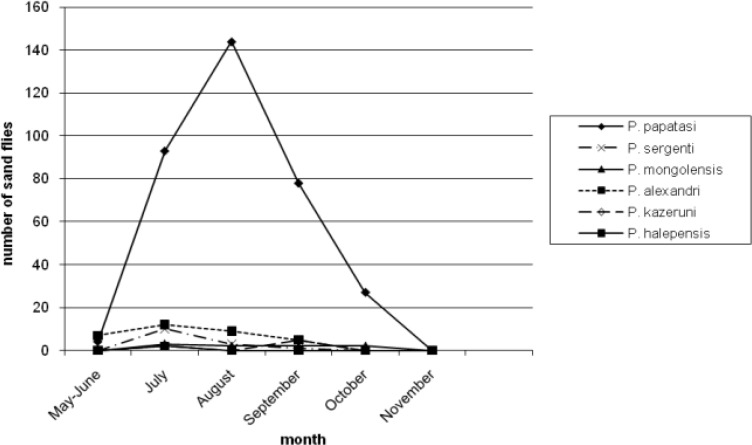
Monthly activity of *Phlebotomus* species from May to November caught from outdoor walls

**Fig. 5:**
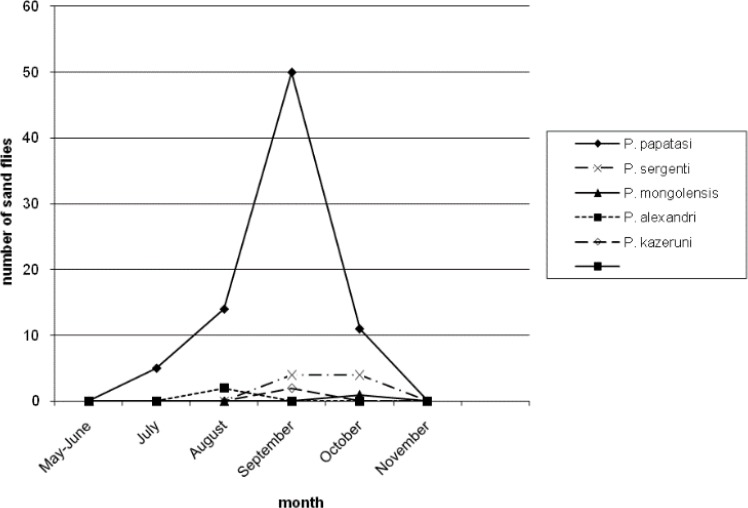
Monthly activity of *Phlebotomus* species from May to November caught from warehouses

Table 3 reports number of *Phlebotomus* species collected from different resting places. Moreover, 46.38% and 22.52% of the population of *P. papatasi* were caught from outdoor walls and human dwellings inside the villages, respectively. Only 2.02% of the population of *P. papatasi* was captured from mountains, outside the villages. Four species (*P. major, P. halepensis, P. jacusieli and P. elenorae*) had the lowest population in the area (Table 3). The monthly activity of sand flies in the area was from May to Nov and the peak of their activities was during July and Aug (Fig. 1–5).

**Table 3:** Prevalence of *Phlebotomus* species caught from resting places

**Species**	**Human dwelling**	**Animal dwelling**	**Mountain**	**Outdoor walls**	**Ware houses**	**Total**
*P. papatasi*	168 (22.52)	137 (18.36)	15 (2.02)	346 (46.38)	80 (10.72)	746
*P. sergenti*	1 (2.86)	0 (0.00)	12 (34.29)	14 (40.00)	8 (22.85)	35
*P. alexandri*	11 (13.93)	17 (21.52)	14 (17.72)	33 (41.77)	4 (5.06)	79
*P. elenorae*	5 (83.33)	1 (16.67)	0 (0.00)	0 (0.00)	0 (0.00)	6
*P. jacusieli*	0 (0.00)	3 (100.00)	0 (0.00)	0 (0.00)	0 (0.00)	3
*P. major*	1 (100.00)	0 (0.00)	0 (0.00)	0 (0.00)	0 (0.00)	1
*P. kazeruni*	12 (52.17)	2 (8.70)	0 (0.00)	7 (30.43)	2 (8.70)	23
*P. halepensis*	0 (0.00)	0 (0.00)	0 (0.00)	2 (100)	0 (0.00)	2
*P. mongolensis*	0 (0.00)	2 (11.77)	5 (29.41)	9 (52.94)	1 (5.88)	17
Total	198	162	46	411	95	912

Fig. 1 shows the monthly activity of *Phlebotomus* genus caught from animal dwellings. The peak of population of *P. papatasi* and most species in the area is in Aug with a sharp increase from May and sharp decrease up to Nov. The pattern of the monthly population of *Phlebotomus* genus according to place of the collection have been shown in Fig. 2–5 for human dwellings, outdoor shelters (mountain), outdoor walls inside the villages and ware houses, respectively.

The dominant species and main vector of disease in the area was *P. papatasi*. Due to the latency period of this disease and the highest activity of the vector during spring and summer, we expected emerging of most cases of disease at autumn and winter.

### Comparison of efficiency of ITNs and IRS in reducing the cases of CL

In the beginning of the study, the incidence of disease was 4651.2 per 100000 population of the area (178 positive cases), while 60.3% of reported cases were female and 39.7% male. Most cases of disease belonged to the age group of 10 to 20 yr old (43 cases) and the last belonged to the oldest ages (> 50).

After 1 yr of trial, the cases of disease reduced from 50 to 2 and 128 to 5 for ITNs distributed nets and using of IRS in the villages, respectively. Reduction of cases in both control methods was significant (*P*<0.001). The difference between 2 methods was not significant (*P*>0.05). Cost-benefit analysis of expenses of 2 methods showed that the expenses of using ITNs are about 61% of IRS method.

## Discussion

CL is a public health problem at a global level affecting 1.5 million people annually worldwide. “This disease characterized by chronic skin lesions and leaves permanent scars as the deformation of the infected area” (1).

Most sand flies caught in different places in the present study belonged to *Phlebotomus* genus (912) in comparison to *Sergentomyia* (152). The results showed that the population and duration of activity (May to Nov) of *Phlebotomus* genus in the area for transmission of disease is high and *P. papatasi* is the dominant and main vector of CL in the area.

The activity of *Phlebotomus* genus in the area starts from May and the population of sand flies increases up to Aug and then decreases up to Nov. Finding of both control methods showed that they had the same efficacy in reduction of CL, but ITNs control method was cheaper than IRS. Meanwhile, IRS method pollutes the environment regards to mass usage of insecticide. Similar projects on control of malaria have reported similar findings of our investigation. In a trial in China the effect of insecticide treated bed nets and DDT residual spraying on the prevalence of malaria were compared. Both methods were equally effective, but deltamethrin treatment of nets was cheaper and so was chosen for vector control (24). In Western Thailand (25), the cost of DDT spraying was 2.4 times higher than that of net impregnation. In the warmer parts of China, the use of bed nets against malaria is already very widespread and it was reported to be cheaper to treat the nets in a house with delta-methrin annually (15 mg/m^2^) than to spray the same house with DDT (26).

Health center of the county started IRS of villages after the outbreak of CL during the transmission season using deltamethrin Emulsion, 2.5% EG (20–25 cc in 20 L water) or Aicon 10WP, 2 sachets (62.5 gr) in 18–20 L water (active ingredient 25–30 mg/m2 of sprayed surface). IRS method of control started two years before starting the present study, but the incidence of disease did not reduce. One of the reasons maybe was due to using the wrong method of spraying by workers. Training of sprayer workers is an important issue in CL control using IRS method. Before starting the study, the workers started to spray against sand flies from 2 yr ago believed that sunlight degrades the insecticide so they did not spray the outdoor walls and other spaces exposed to sunlight that were potential habitats of sand flies. This is maybe an important reason that why IRS did not work well in reducing of disease before starting of the present study. Another reason maybe was that workers did not spray inside of the holes in outdoor walls and other potential habitats of sand flies. Therefore, during spraying of four villages selected in the study for IRS, workers were trained for correct method of spraying of walls.

Synthetic pyrethroids are designed not to be photo-liable in this way but one anticipates some degree vulnerability to bright sunlight (27–29). In another study by the International Panel on Chemical Safety, the researchers found many variations in half-life of deltamethrin in the field according to conditions (30). Nevertheless, the reported half-lives in such studies are generally measured in weeks (not days). FAO/WHO, Meister, and Worthing stated that “deltamethrin is stable to heat (six months at 40 °C), light and air, but unstable in alkaline media “ (31–33).

Previous investigations in Iran (15, 17), Syria (16), Turkey (14), and Afghanistan (13), showed that ITNs are effective against CL. ITNs, chaddars or top sheets, and residual spraying of interior rooms were effective tools for the prevention of CL in Kabul, Afghanistan (13). Four studies carried out in Isfahan Province in the South-West of Iran (2, 15), northeastern Iran (17), Shiraz city (2) and Bam in southeastern Iran (34, 35) reported the efficacy of ITNs in reducing of CL.

We believe that health education and training people about transmission and prevention of diseases are very important tools in control of diseases. We asked people not to stay outdoors and asked women to wash their utensils indoors after evening. Most households have cheap cooling systems at their homes, so after evening they can stay and sleep indoors during warm seasons.

Nets were distributed between householders only in 2 d, although to complete IRS in other villages approximately 3 wk timetable was necessary. This is an important point in saving time and rapid prevention of disease when it outbreaks if ITNs are used.

## Conclusion

ITNs is a cheap, safe, easy to use and rapid tool for prevention and control of CL.

## Ethical considerations

Ethical issues (including plagiarism, informed consent, misconduct, data fabrication and/or falsification, double publication and/or submission, redundancy, etc.) have been completely observed by the authors.
